# Changes in Alcohol Consumption Pattern Based on Gender during COVID-19 Confinement in Spain

**DOI:** 10.3390/ijerph18158028

**Published:** 2021-07-29

**Authors:** Víctor J. Villanueva-Blasco, Verónica Villanueva Silvestre, Manuel Isorna, Patricia Motos, Pere Blay, Andrea Vázquez-Martínez

**Affiliations:** 1 Faculty of Health Sciences, Valencian International University, 46002 Valencia, Spain; avazquezm@universidadviu.com; 2 Faculty of Education and Social Work, Campus As Lagoas, University of Vigo, 32004 Ourense, Spain; isorna.catoira@uvigo.es; 3 Faculty of Psychology, University of Valencia, 46010 Valencia, Spain; patricia.motos@uv.es; 4 School of Science and Technology, Valencian International University, 46002 Valencia, Spain; pjblay@universidadviu.com

**Keywords:** alcohol, risk consumption, standard drink unit (SDU), COVID-19, confinement measures, gender

## Abstract

(1) The goal of this study was to analyze the prevalence and pattern of alcohol consumption (frequency of consumption, average daily consumption, and risky consumption) before and during confinement due to the coronavirus disease (COVID-19) in the adult population and based on gender. (2) Methods: Data from 3779 individuals were collected via a set of online surveys. The AUDIT alcohol consumption questions (AUDIT-C) were used to measure the frequency of consumption, the average daily consumption, intensive consumption, risky consumption, and standard drink units. (3) Results: During confinement, the prevalence of alcohol consumption declined in both males and females, but only intensive consumption showed significant differences, with a greater reduction in males. The number of females who consumed alcohol four or more times per week doubled, whereas the number of males who did so was multiplied by a factor of 1.5; in both females and males, the percentage who presented intensive consumption doubled. The percentage of females with risky consumption was higher than that of males both before and during confinement. In addition to gender, the interaction between age and the employment situation explain consumption before and during confinement. (4) Conclusions: During confinement due to COVID-19, alcohol consumption declined in both sexes, but alcohol-risk consumers increased their frequency of use. The interaction between gender, age, and employment situation was related to these changes. These findings are relevant for guiding public health and health-risk management policies related to alcohol consumption in environmental situations similar to COVID-19.

## 1. Introduction

The COVID-19 pandemic made it necessary to adopt measures to contain its spread and effects on the population’s health. Confinement measures and mobility restrictions resulted in lifestyle changes, isolation, restricted social contact, loss of freedom, and closure of nonessential businesses. Places where alcohol was regularly consumed (bars, restaurants, clubs, pubs), especially by young people [1], were closed. However, as referenced by these authors, Internet sales of alcoholic beverages increased 240% during this period. In Spain, a 24.7% increase in alcohol in shopping baskets was observed in April 2020 compared to March 2019 [2].

The application of these restrictive measures led to a number of changes that had an impact on mental health [3], such as increases in psychosocial stress [4,5] and anxiety [6]. Due to its effects on the nervous system, alcohol can be used as a tool to mitigate unpleasant emotions, stress, anxiety, or depression [7,8].

Studies on alcohol consumption during the COVID-19 pandemic show contradictory results. Some studies noted that, compared to consumption levels before the pandemic, the prevalence of alcohol consumption, consumption frequency, number of drinks on each occasion, or intensive consumption episodes stayed the same or decreased during confinement, mainly in the young population in the 18- to 29-year-old age range [9,10]. In contrast, some studies indicated an increase in consumption during the same period [11,12,13,14].

Few studies have been carried out on the gender differences in alcohol consumption during confinement. Villanueva [10] noted that males showed higher scores than females on the AUDIT-C before and during confinement. Another study by Pollard [15] found a 17% increase in alcohol consumption in females, whereas other studies related the excessive consumption of alcohol to the high levels of anxiety associated with COVID-19 in females but not in males [16]. Moreover, it should be taken into account that due to the confinement measures and mobility restrictions, alcohol consumption was limited to the home. In this regard, some studies indicate that, contextually, drinking alcohol in the home is more associated with females than with males, and it is a strong predictor of risky consumption in females [17,18,19]. It is certainly necessary to thoroughly examine gender differences in order to obtain a more detailed picture.

Other variables that may have been related to changes in alcohol consumption during confinement are worth mentioning. For example, regarding age, some studies indicate that alcohol consumption was reduced to a greater extent in young adults than in older adults [1,10,20]; moreover, in economic crisis situations, some people reduce their alcohol consumption due to financial problems or the risk of losing their job if they continue to drink to excess [21]. These sociodemographic variables could interact with the “gender” variable. Some studies [22,23] have pointed out that drinking patterns in females have changed in recent years, especially in younger age groups. In addition, due to gender inequalities in the workplace and the way the COVID-19 pandemic containment measures influenced them, differences in the consumption patterns of females and males might exist.

The goal of this study was to analyze whether changes in the prevalence and pattern of alcohol consumption (frequency of consumption, daily average consumption, intensive consumption, standard drink units, and risky consumption) in the Spanish adult population varied based on gender during confinement, compared to previous time periods. The possible interaction between gender and age and the employment situation before and after confinement were analyzed.

## 2. Materials and Methods

### 2.1. Design

This study was descriptive and nonprobabilistic, and it used convenience sampling. A battery of online surveys was used to collect and evaluate the variables under study. Age ranges were established based on those that showed adequate Internet access, as stated in the Equipment and Use of Information and Communication Technologies at Home Survey [24].

### 2.2. Population

The initial sample included 4213 participants. Of them, 434 (10.3%) were removed because of missing values, incoherent response patterns, or being outside the established age range (18–64 years old). The final sample contained data from 3779 participants (70% female; 30% male) with an average age of 37.76 years (SD = 11.95), corresponding to 17 autonomous regions and the two Spanish autonomous cities. By age range, 14.8% (*n* = 558) were 18–24 years old, 17.3% (*n* = 656) were 25–29 years old, 13.8% (*n* = 522) were 30–34 years old, 23.8% (*n* = 900) were 35–44 years old, 19% (*n* = 717) were 45–54 years old, and 11.3% (*n* = 427) were 55–64 years old. Regarding employment, 47.4% (*n* = 1142) had a full-time job, 8.4% (*n* = 203) had a part-time job, 7.7% (*n* = 186) were self-employed, 9.7% (*n* = 235) had a job covered by a Temporary Employment Regulation Plan (ERTE), 1% (*n* = 23) were homemakers, 14.7% (*n* = 355) were students, 1.3% (*n* = 31) were pensioners or retirees, 8.9% (*n* = 215) were unemployed, and 0.9% (*n* = 21) chose to leave this question blank.

The database was weighted to correct for the bias introduced due to the intentional nonprobabilistic nature of the sampling, which translated into a sampling imbalance in the gender of the participants.

### 2.3. Procedure

Data collection started on 14 April 2020, after the first 30 days of confinement measures, and it ended on 29 May, when the de-escalation measures started. The data collection strategy was based on a survey hosted on the web, posts on social media, and advertisements via e-mail and smartphone messaging applications. Participants were informed that participation was voluntary, in accordance with the Spanish Organic Law 3/2018 of Personal Data Protection and Digital Rights Guarantee [25]. They were asked to give their consent to participate. Selection criteria were as follows: (a) age between 18 and 64; (b) explicit agreement to participate; and (c) properly filling out the survey.

### 2.4. Study Variables

The sociodemographic variables considered were: (a) gender (male, female); (b) age, according to the age ranges established in the EDADES survey [26] (18–24 years, 25–29 years, 30–34 years, 35–44 years, 45–54 years, 55–64 years); and (c) employment status during confinement: (1) full-time employment, (2) part-time employment, (3) self-employed, (4) being under an employment regulation plan, (5) homemakers, (6) students, (7) pensioners or retirees, and (8) unemployed.

AUDIT-C [27], a short version of the Alcohol Use Disorders Identification Test, was used to measure alcohol consumption. AUDIT-C is composed of three items that analyze consumption frequency, average daily consumption, and frequency of intensive consumption. Frequency of consumption (average consumption days per month) was measured with the question “How often do you consume alcoholic drinks?”, with possible answers being (0) “Never”, (1) “Once a month or less”, (2) “2 to 4 times per month”, (3) “2 to 3 times per week”, or (4) “4 or more times per week”. Daily average consumption was measured with the question “How many alcoholic drinks do you usually have on a normal day?”, with possible answers being (0) “1 or 2”, (1) “3 or 4”, (2) “5 or 6”, (3) “7, 8, or 9”, and (4) “10 or more”. Intensive consumption was measured with the question “How often do you drink 6 or more alcoholic beverages in a single day?”, with possible answers being (0) “Never”, (1) “Less than once a month”, (2) “Monthly”, (3) “Weekly”, (4) “Daily or almost daily”.

Risky consumption is defined as a pattern of consumption that increases the risk or likelihood of harmful consequences for the consumer, even when the consumer does not have any current disorders [28]. The limit of risky consumption was established at 4 points or more in females and 5 or more in males, based on the total score on the AUDIT-C [29,30].

Furthermore, the Spanish Standard Drink Unit (SDU), according to which 1 fermented beverage (beer, wine) = 1 SDU; and 1 distilled beverage (spirit, liquor) = 2 SDUs [31], was used. Because this is a standard measure, the amount of alcohol ingested in a day can be recorded more accurately. A Likert-type response scale with six options was used: (0) 1 or 2; (1) 3 or 4; (2) 5 or 6; (3) 7, 8, or 9; and (4) 10 or more. Participants were given exact information about the SDU equivalencies.

Participants were asked about these drinking variables in relation to the confinement period (April–May 2020) and retrospectively in relation to their drinking in the six months prior to the pandemic (March 2020).

### 2.5. Statistical Analysis

Data analysis was performed with the IBM SPSS Statistics for Windows, version 25 (IBM Corp., Armonk, NY, USA). As a first step, the sample was weighted as a balancing strategy. After that, intragroup differences were examined through a frequency analysis and chi-square test (disaggregated according to gender) of the frequency of consumption rate, average daily consumption, intensive consumption, and SDUs per day before and during confinement. To compare measures of the cited variables, as well as the score on the AUDIT-C to establish the alcohol consumption before and during the pandemic, compliance with the normality criterion (Kolmogorov–Smirnov) and homoscedasticity (Levene’s equal variances) was checked, considering gender as the independent variable, by applying a Student’s *t*-test.

Comparisons of between-group means before and during confinement were also carried out. For independent samples, a Student’s *t*-test was performed to analyze differences between females and males. To obtain a measure of the effect size, *Phi* and Cohen’s d was used.

Finally, several analyses of variance (ANOVA) were performed to study the interaction effects between the three AUDIT-C variables and the SDU measurement before and during confinement and the gender variable, subsequently including age and employment status.

### 2.6. Ethical Aspects

The study was carried out in accordance with the Code of Ethics of the World Medical Association (Declaration of Helsinki) and approved by the Committee of Evaluation and Follow-up of Research with Human Beings (CEISH) from Valencian International University (VIU).

## 3. Results

Of the total sample (*n* = 3779), 62% of the participants (*n* = 2345) had consumed alcohol in the past six months; 46.65% of them (*n* = 1094) were female, and 53.35% (*n* = 1251) were male. Table 1 shows prevalence data for the) frequency of consumption rate, average daily consumption, intensive consumption, and average SDUs per day both before and after confinement, depending on gender.

An increase in the prevalence of nonconsumption was seen in both females (10 times greater) and males (8 times greater) during confinement compared to before the pandemic. A change during confinement was also observed in intensive alcohol consumption, which increased 22.4% in females and 28.2% in males who did not show intensive consumption before confinement.

Moreover, during confinement, a decrease in the prevalence was noted on all the daily average consumption indicators, except 1 to 2 drinks per day, where there was an increase in the prevalence. This was observed in both genders, with an increase of more than 10% in consumers of 1 to 2 drinks per day, and a 2- to 3-fold decrease in consumers of 5 or more alcoholic drinks per day.

In general, during confinement, the prevalence decreased for all the SDU per day indicators, except 1–2 SDUs, where the prevalence increased for both genders.

However, during confinement, the number of females who consumed alcohol four or more days per week doubled, whereas it was only 1.5 times greater for males. With regard to intensive consumption, the percentage of both males and females who presented this consumption pattern doubled during confinement compared to before it.

The analysis of the average differences in the frequency of consumption rate (Table 2) showed significant differences before (M = 2.26; SD = 0.969) and during confinement (M = 1.98; SD = 1.355; t_(2343)_ = 13.170; *p* < 0.001). The same situation was observed for average daily consumption, with a higher number of alcoholic drinks per day consumed before confinement (M = 0.26; SD = 0.586) than during confinement (M = 0.12; SD = 0.404; t_(2343)_ = 12.318; *p* < 0.001), and with intensive consumption being more frequent before confinement (M = 0.63; SD = 0.886) than during it (M = 0.27; SD = 0.709; t_(2343)_ = 21.429; *p* < 0.001). Regarding the SDUs consumed per day, higher numbers were found before confinement (M = 0.17; SD = 0.507) than during confinement (M = 0.12; SD = 0.450; t_(2343)_ = 4.637; *p* < 0.001).

**Figure 1:**
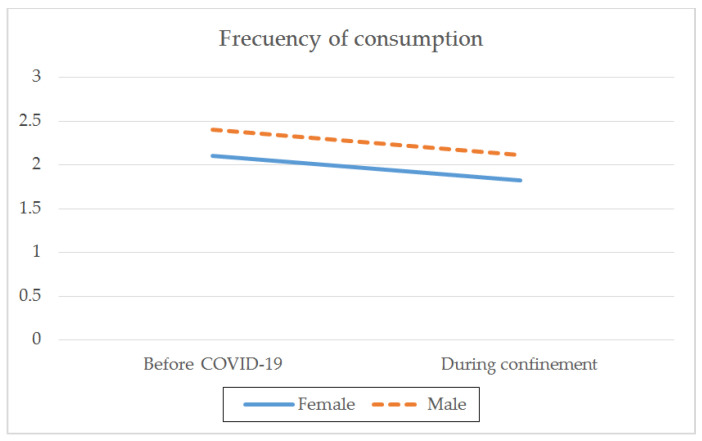
Average frequency of consumption by gender before the pandemic and during COVID-19 confinement.

**Figure 2:**
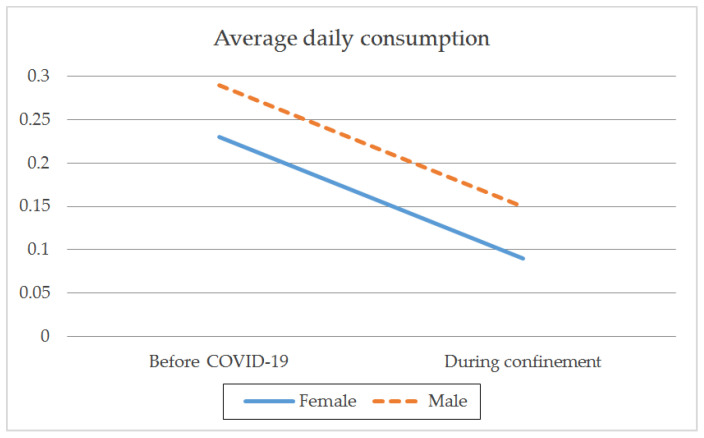
Average daily alcohol consumption by gender before the pandemic and during COVID-19 confinement.

**Figure 3:**
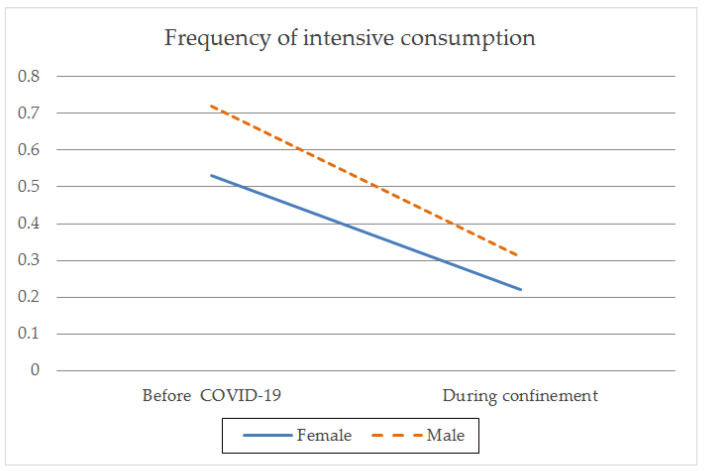
Frequency of intensive alcohol consumption by gender before the pandemic and during confinement due to COVID-19.

**Figure 4:**
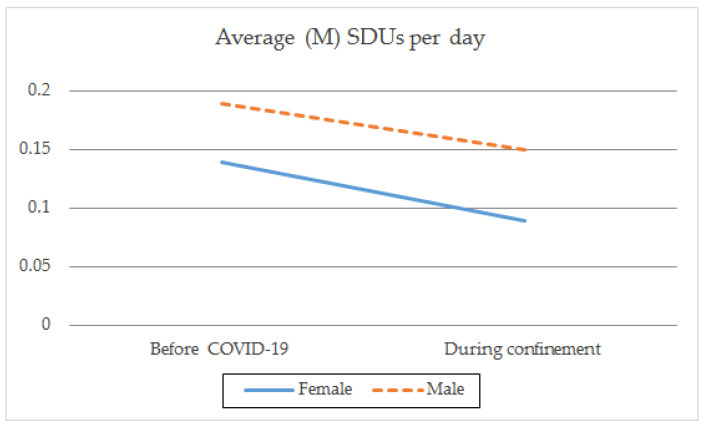
Average number of SDUs per day by gender before the pandemic and during COVID-19 confinement.

With regard to gender, there were both intragroup and between-group differences before and during confinement. Both genders showed a higher frequency of consumption rate (t_(1259)male_ = 9.797; *p* < 0.001; t_(1086)female_ = 8.801; *p* < 0.001) (Table 2; Figure 1), a larger average daily intake (t_(1259)male_ = 8.459; *p* < 0.001; t_(1086)female_ = 9.166; *p* < 0.001) (Table 2; Figure 2), and a greater frequency of intensive consumption (t_(1250)male_ = 17.250; *p* < 0.001; t_(1093)female_ = 12.875; *p* < 0.001) (Table 2; Figure 3) before confinement than during confinement. Males showed a higher frequency of consumption rate than females both before (M_male_ = 2.40; SD = 0.999; M_female_ = 2.10; SD = 0.908; t_(2343)_ = −7.549; *p* < 0.001) and during confinement (M_male_ = 2.11; SD = 1.369; M_female_ = 1.82; SD = 1.323; t_(2343)_ = −5.084; *p* < 0.001) (Table 2; Figure 1). Males also showed a greater average daily consumption both before (M_male_ = 0.29; SD = 0.626; M_female_ = 0.23; SD = 0.536; t_(2343)_ = −2.693; *p* < 0.01) and during confinement (M_male_ = 0.15; SD = 0.446; M_female_ = 0.09; SD = 0.347; t_(2343)_ = −3.723; *p* < 0.001) (Table 2; Figure 2). Males also presented greater intensive alcohol consumption before (M_male_ = 0.72; SD = 0.923; M_female_ = 0.53; SD = 0.830; t_(2340.473)_ = −5.389; *p* < 0.001) and during confinement (M_male_ = 0.31; SD = 0.744; M_female_ = 0.22; SD = 0.665; t_(234.244)_ = −2.996; *p* < 0.01) (Table 2; Figure 3). Both genders showed a larger average number of SDUs per day before confinement than during confinement (t_(1250) male_ = 2.638; *p* < 0.01; t_(1093) female_ = 4.126; *p* < 0.001) (Table 2; Figure 4). Males showed a larger average number of SDUs per day both before (M_male_ = 0.19; SD = 0.539; M_female_ = 0.14; SD = 0.466; t_(2343.133)_ = −2.205; *p* < 0.05) and during confinement (M_male_ = 0.15; SD = 0.507; M_female_ = 0.09; SD = 0.372; t_(2275.708)_ = −3.427; *p* < 0.001) (Table 2; Figure 4).

When analyzing the interaction effect between gender and each of the variables studied, we found that during confinement, males decreased their frequency of intensive consumption to a greater extent than females (F_(1.3028)_ = 13.575; *p* < 0.001) (Table 2). This interaction effect was not observed between gender and the frequency of monthly consumption (F_(1.3028)_ = 0.130; *p* = 0.719), average daily consumption (F_(1.3028)_ = 0.031; *p* = 0.860), or average number of SDUs per day (F_(1.3028)_ = 0.899; *p* = 0.343). This indicates that during confinement, there was a reduction in each of these variables, regardless of the gender.

**Figure 5:**
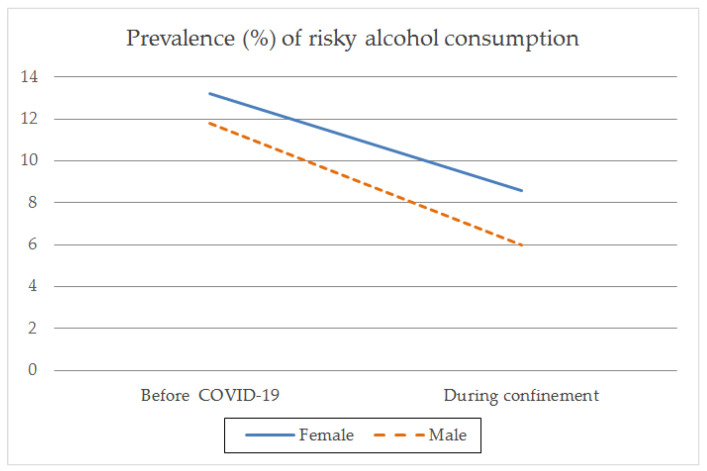
Prevalence (%) of risky alcohol consumption by gender before the pandemic and during COVID-19 confinement.

In relation to risky alcohol consumption, 25.9% (*n* = 608) were classified as risky consumers before the pandemic, decreasing to 15.1% (*n* = 353) during the period of the confinement measures. The proportion of consumers at high alcohol risk was greater in females than in males both before the pandemic (females 29.4%, *n* = 322; males 22.9%, *n* = 286; X^2^ = 13.124; *p* < 0.001; Phi = −0.075) and during confinement (female 19.1%, *n* = 209; male 11.5%, *n* = 144; X^2^ = 26.316; *p* < 0.001; Phi = −0.106), with a very low effect size in both time segments. In relation to the percentage of consumers with high alcohol risk, there was a significant decrease during confinement compared to before the pandemic in both males (X^2^_MN_ = 92.113; *p* < 0.001) and females (X^2^_MN_ = 54.568; *p* < 0.001), with a medium effect size (Table 3; Figure 5).

When analyzing the AUDIT-C scores in the subsample of high-risk consumers and the interaction effects between the sociodemographic variables (age and employment status), the gender variable, and the four alcohol consumption variables before the pandemic and during confinement, an interaction effect was found between the average daily alcohol consumption before and during confinement and gender, age, and employment status (F_(16.661)_ = 3.797; *p* < 0.001). An interaction effect was also found between the frequency of intensive consumption before the pandemic and during confinement and gender, age, and employment status (F_(16.661)_ = 2.249; *p* < 0.01). Finally, an interaction effect was found between the number of SDUs per day before the pandemic and during confinement and gender, age, and employment status (F_(16.661)_ = 2.235; *p* < 0.01).

Based on these results, a descriptive analysis of age and employment status by gender was performed in the subsamples of high-risk consumers according to the AUDIT-C before and during confinement.

In the subsample of females who presented risky alcohol consumption, the mean age was 33.38 (SD = 11.18) before confinement and 39.06 (SD = 11.31) during confinement. With regard to employment, they were mostly full-time employees (37.3%_before_; 47.1%_during_), followed by students (20.4%_before_; 8.9%_during_), part-time employees (11.1%_before_; 7.2%_during_), ERTE-regulated employees (10.97%_before_; 8.2%_during_), unemployed (10.2%_before_; 13.3%_during_), self-employed (6.7%_before_; 9.9%_during_), pensioners or retirees (0.7%_before_; 1.7%_during_), and homemakers (0.7%_before_; 1.7%_during_).

In the subsample of males who presented risky alcohol consumption, the mean age was 36.81 (SD = 11.83) and 40.85 (SD = 11.62) before and during confinement, respectively. With regard to their employment situation, the majority were full-time employees (45%_before_; 46.5%_during_), followed by students (12.3%_before_; 3.5%_during_), part-time employees (10.5%_before_; 9.3%_during_), ERTE regulated employees (10.5%_before_; 18.6%_during_), self-employed (9.4%_before_; 10.5%_during_), unemployed (9.4%_before_; 9.3%_during_), pensioners or retirees (1.8%_before_; 2.3%_during_), and homemakers (1.2%_before_; 0%_during_).

## 4. Discussion

The goal of this study was to evaluate whether the prevalence and patterns of alcohol consumption changed during confinement due to COVID-19 compared to before the pandemic. The lack of studies focusing on the modification of alcohol consumption patterns during confinement based on gender makes this study a reference. In addition, the results suggest that the changes observed in the pattern of alcohol consumption in both genders are equally modulated by age and employment status. These findings make an important contribution to better understanding the relationships between these variables and different drinking patterns.

Our results show that alcohol consumption declined during confinement, in contrast to the results of other studies [11,12,13,14]. We found fewer alcohol consumers, a lower consumption frequency and intensive consumption rate, and smaller amounts in both average daily consumption and in the number of SDUs per day. This effect was common to both genders, although it is more pronounced in females. Our finding contradicts the common assumption that alcohol consumption would increase during confinement, as the increase in sales of alcoholic drinks in home shopping baskets [2] and online [1] would suggest. However, this increase in alcoholic drinks in shopping baskets alone is not a reliable measure of alcohol consumption because it does not account for the decrease in consumption outside the home [32].

During confinement, both genders showed a decrease in the frequency of consumption and intensive consumption, quantities, and SDU per day. However, significant differences were found only for males in the frequency of heavy drinking (6 or more alcoholic beverages on the same day), showing a greater decrease compared to females. This finding suggests that mobility restrictions and home confinement measures had an impact on the frequency of heavy drinking patterns in males, but not in females. Thus, the drinking contexts associated with heavy drinking patterns may be different for the two genders, with males drinking outside the home and females drinking more in the home. In this regard, some studies point to an increase in alcohol consumption in the home in females [17,18,19], which would corroborate our findings.

Furthermore, we also found that a subset of the population of alcohol consumers increased their frequency of consumption rate. During confinement, the number of females who consumed alcohol four or more times per week doubled, whereas in males it was multiplied by a factor of 1.5; in both females and males, the percentage of people with a heavy drinking pattern doubled. In other words, both genders showed that part of the adult population increased the frequency of their alcohol consumption and heavy drinking, which is consistent with other findings indicating that part of the drinking population showed no changes or reduced their alcohol consumption, whereas another part showed an increase in alcohol consumption [33]. However, the increase in the percentage of females with a higher frequency of alcohol consumption (four or more times per week) was greater than that of males. We examined the percentage of females who presented risky alcohol consumption according to the AUDIT-C, with 3 out of 10 females consuming at this level before the pandemic and 2 out of 10 during confinement, compared to 2 and 1 out of 10 males, respectively. This finding is in line with what was observed by Pollard [15]. In addition, as pointed out by several authors [18,19], the risk increases exponentially when, in addition to daily home consumption, alcohol is purchased as part of the weekly shopping and there is a greater preference for drinking at home than in public places [18,19]. Another issue to consider is that the effects of alcohol consumption occur more rapidly and last longer in females than in males after drinking equivalent amounts, with females reaching higher blood alcohol levels [34,35]. Moreover, females with alcohol consumption habits visit treatment centers less frequently and take longer to visit them, which hinders their therapeutic care [36].

When analyzing the effect of the interactions between the sociodemographic variables (sex, age, educational level, and employment status) along with the four alcohol consumption variables related to before the pandemic and during confinement, we observed that in all cases, there was a decrease in the frequency of alcohol consumption, the average amount, intensive consumption, and SDUs during confinement compared to before confinement. However, this decrease differed depending on the interactions between the sociodemographic variables, which suggests a much more complex relationship between these variables.

In the case of age, according to the AUDIT-C, in the subsample of risky alcohol consumers, an increase in the mean age was observed in both females and males during confinement compared to before confinement. In females, this increase was 5.68 years, and in males 4.04 years. This finding was related to the decrease from 20.4% to 8.9% (2.23/1) in females and from 12.3% to 3.5% (3.51/1) in males observed in the subsample of students with risky drinking before and during confinement. Compared to older drinkers, alcohol consumption among younger drinkers occurs mainly outside the home [37,38], and so it is likely that younger drinkers decreased their consumption due to the closure of nightlife (bars and discotheques) and the impossibility of drinking in public. Different studies [1,10,20,39] have shown that in the younger population (18–25 years), the prevalence of risky consumption decreased more significantly than in older people.

With regard to the changes observed in relation to employment status in the subsample of risky consumers, there was an increase in the prevalence of females in full-time employment, from 37.1% to 47.1%, whereas in males there were no changes. A possible explanation is that females are harmed by inequity in home and family duties, which complicates the work–family balance during confinement [40] and can lead to the use of alcohol to release stress and anxiety caused by the pandemic situation [16]. Another noteworthy finding is that the prevalence of females with risky alcohol consumption decreased by around 2.8% in ERTE-regulated employees, whereas in males it increased by 8.1%. In contrast, among self-employed and unemployed people, there was an increase of about 3.2% in the prevalence in females in both situations, while it remained very similar in men. These findings show the complex relationships between these variables, as well as the need to deepen our understanding through additional studies. This is especially relevant because some studies [17] indicate that people with a lower socioeconomic level consume more alcohol on each occasion, which would imply a higher risk of mortality in lower socioeconomic groups.

## 5. Conclusions

Although alcohol consumption decreased during confinement due to COVID-19, as did the prevalence of risky consumers in the population, a large part of the population showed risky consumption patterns both before and during confinement. Likewise, during confinement there was an increase in the prevalence of heavy drinkers. It is possible that those who mostly drank alcohol outside the home may have decreased their consumption due to the restrictions, whereas drinkers with a more frequent and intensive drinking pattern may have increased their consumption. Although these findings were observed in both females and males, significant differences were found only in males in the frequency of heavy drinking. The rest of the changes in the consumption pattern were explained by the interaction with sociodemographic variables such as gender, age, and employment status.

Our findings must be considered when designing alcohol consumption prevention strategies and policies, which must include the gender perspective and social equity as factors in order to reach the most vulnerable collectives and ensure their effectiveness.

Some of the limitations of this study are the possible errors in coverage, the randomness of the sample, and the response rate, due to the use of an online survey. In any case, actions to compensate for these errors were carried out (see Design and Population sections above). Although our sample was large, it cannot be considered representative of the Spanish population. Therefore, the findings should be generalized with caution. Moreover, it is necessary to consider the possible cultural differences in the cut-off points for risky alcohol consumption based on gender in the different countries. This limits the extrapolation of the results. The fact that all the variables were self-reported may lead to various biases due to under- or overestimation, especially when retrospectively asking about alcohol consumption during the six months prior to the pandemic. Longitudinal studies would allow us to find out whether this change lasts over time.

## Figures and Tables

**Table 1:** Prevalence of the frequency of consumption rate and average daily intake of alcohol before and during confinement, as a function of gender.

		Before Confinement		During Confinement	
		Female % (*n*)	Male % (*n*)	Female % (*n*)	Male % (*n*)
Frequency of consumption	Never	2.67 (29)	1.99 (25)	20.72 (225)	16.04 (202)
	Once or twice per month	21.82 (237)	16.04 (202)	22.47 (244)	19.14 (241)
	2–4 times per month	45.76 (497)	36.93 (465)	25.04 (272)	22.95 (289)
	2–3 times per week	23.39 (254)	28.83 (363)	18.69 (203)	20.33 (256)
	4 or more times per week	7.09 (77)	15.57 (196)	13.81 (150)	20.89 (263)
Average daily consumption	1 or 2	82.50 (896)	77.84 (980)	93.64 (1017)	87.85 (1106)
	3 or 4	14.18 (154)	15.57 (196)	5.52 (60)	8.74 (110)
	5 or 6	3.22 (35)	4.37 (55)	1.29 (14)	2.38 (30)
	Between 7 and 9	0.83 (9)	1.59 (20)	0.27 (3)	0.40 (5)
	10 or more	0 (0)	0 (0)	0 (0)	0 (0)
Intensive consumption	Never	64.4 (704)	52.7 (659)	86.8 (949)	80.9 (1012)
	Less than once a month	23.3 (255)	29.5 (370)	8.3 (91)	11.8 (147)
	Monthly	8.1 (88)	11.2 (140)	1.8 (19)	4.1 (52)
	Weekly	3.8 (42)	6.0 (75)	2.3 (26)	2.1 (27)
	Daily or almost every day	0.4 (4)	0.5 (7)	0.8 (9)	1.1 (13)
Average SDUs ^1^ per day	1 or 2	89.1 (975)	86 (1075)	93.2 (1020)	89.3 (1117)
	3 or 4	8.3 (91)	10.6 (132)	5.3 (58)	7.8 (97)
	5 or 6	1.8 (19)	2.4 (30)	0.9 (10)	2 (25)
	Between 7 and 9	0.6 (6)	0.7 (8)	0.4 (4)	0.4 (5)
	10 or more	0.2 (2)	0.4 (5)	0.1 (1)	0.5 (7)

^1^ Standard Drink Units.

**Table 2:** Alcohol consumption patterns before and during confinement as a function of gender.

		Before Confinement M (SD ^1^)	During Confinement M (SD ^1^)	*t*	*p*	*d*
Frequency of consumption	Female	2.10 (0.908)	1.82 (1.323)	8.801	0.001	−0.351
	Male	2.40 (0.999)	2.11 (1.369)	9.797	0.001	−0.343
Average daily consumption	Female	0.23 (0.536)	0.09 (0.347)	9.166	0.001	−0.242
	Male	0.29 (0.626)	0.15 (0.446)	8.459	0.001	−0.205
Frequency of intensive consumption	Female	0.53 (0.830)	0.22 (0.665)	12.875	0.001	−0.362
	Male	0.72 (0.923)	0.31 (0.744)	17.250	0.001	−0.443
Average SDUs ^2^ per day	Female	0.14 (0.466)	0.09 (0.372)	4.126	0.001	1.565
	Male	0.19 (0.539)	0.15 (0.507)	2.638	0.008	−0.075

^1^ Standard Deviation^; 2^ Standard Drink Units.

**Table 3:** Proportion of risky alcohol consumption by gender before and during confinement.

	*n*	Before Confinement % (*n*)	During Confinement % (*n*)	X^2^_MN_^1^	*p*	*Phi*
Female	1094	29.4 (322)	19.1 (209)	54.568	0.001	0.443
Male	1251	22.9 (286)	11.5 (144)	92.113	0.001	0.424

^1^ McNemar’s test.
